# Birthweight and urinary incontinence after childbirth: a systematic review and meta-analysis

**DOI:** 10.1016/j.eurox.2020.100115

**Published:** 2020-09-04

**Authors:** Stian Langeland Wesnes, Elin Seim

**Affiliations:** Research Group for General Practice, Department of Global Public Health and Primary Care, University of Bergen, Norway

**Keywords:** UI, urinary incontinence, OR, odds ratio, CI, confidence interval, birthweight, childbirth, postpartum, puerperium, urinary incontinence

## Abstract

Birthweight and urinary incontinence after childbirth: a systematic review and meta-analysis

Stian Langeland WESNES, Elin SEIM MD

Urinary incontinence (UI) is common after childbirth. Many cohort and cross-sectional studies have reported data on birthweight, but results have not been pooled. It is unclear how birthweight affects UI after childbirth. The objective is to review the effect of birthweight on UI after childbirth through meta-analyses.

Searches were performed in Medline, Embase, Svemed+, ClinicalTrials.gov, Cochrane, and Cinahl in August 2016. Additional reference checking was performed. Included articles evaluated birthweight as a possible risk factor for maternal UI. We included articles that were presented in Norwegian, Danish, Swedish, or English. Two independent reviewers extracted the data and analysed it using Review Manager 5.3 software. Available data from included studies on birthweight (≥4000 g and ≥3500 g, respectively) and UI were combined in meta-analyses. PRISMA and MOOSE guidelines were used.

Eighteen studies (N = 30 070) reported data on birthweight >4000 g vs <4000 g. Birthweight>4000 g compared to weight <4000 g was associated with a significantly increased OR of any UI (OR 1.49, 95% CI 1.24 – 1.80). Five studies (N = 15 066) reported data on birthweight >3500 g vs <3500 g. Birthweight>3500 g was also associated with a significantly increased OR of UI (OR 1.26, 95% CI 1.15 – 1.37).

High birthweight appears to increase OR of UI after childbirth. Preventative strategies should be targeted towards women at particular risk.

## Highlights

• High birthweight significantly increases risk of urinary incontinence after delivery.
• Increasing birthweight (>3500 and >4000 g) leads to increasing risk of incontinence.
• The association is also found in separate analyses on vaginal delivery, primiparous, and stress UI.

## 1 Introduction

Urinary incontinence (UI) is a common problem after childbirth. Prevalence estimates vary from 14 – 45% [1]. A systematic review reported pooled prevalence of any UI to be 32-36% three months postpartum [2]. Reviews on epidural [3], episiotomy [4], cesarean section [2] and instrumental childbirth [5] have clarified their association with UI postpartum. There is inconsistency in the literature regarding remaining birth parameters. By identifying significant risk factors for UI among women after childbirth, future research can identify and validate preventive measures that can be targeted towards these women. Many cohort and cross-sectional studies have reported data on birthweight, but results have not been pooled. In studies using electromyography heavier babies have been associated with evidence of pudendal nerve damage in the pelvic floor after vaginal birth [6], with uncertain clinical significance. The objective of this study was to review the literature to identify studies reporting on the association of high birthweight on urinary incontinence after all modes of childbirth, and to perform meta-analyses on the association of high birthweight on UI after childbirth. If birthweight can be isolated as a risk factor for postpartum urinary incontinence, patients at particular risk can be identified.

## 2 Material and methods

Literature searches were done in Medline, Embase, Svemed+, and Cinahl. Additional search was done in ClinicalTrials.gov and Cochrane Database of Systematic Reviews. A librarian from the University in Bergen assisted in the search in May 09. 2014 and August 24. 2016. Additional search was done May 03. – 14.2017. The search included the following MESH terms and free text; urinary incontinence, leak, urine, bladder, delivery, obstetric, postpartum, postpartum period, puerperium, birthweight, infant, new-born, large, small, SGA, LGA. Abstracts and articles in Norwegian, Danish, Swedish, or English were considered. Both conference abstracts and full publications were included. Additional literature was added based on authors’ knowledge and after reading references in identified literature. Grey literature was not identified.

In the four-part PICO question for this systematic review, we compared women who gave birth with birthweight >4,000 g or >3,500 g, to women giving birth with birthweight <4,000 g or <3,500 g. The outcome was any UI, and stress UI after childbirth.

Search was done in headings and abstracts. Birthweight was sometimes one of several risk factors included in sub-analysis in papers, often not presented in the abstract, and thereby not found by the search strategy. Additional articles were added based on the authors’ knowledge of relevant literature, and after reading references. Identified literature was reviewed separately by both authors. Articles evaluating obstetric risk factors for maternal urinary incontinence in title or abstract were reviewed in full by both authors. When discrepancies between the two authors occurred (Seim, Wesnes), the article was discussed with a third researcher (Rortveit, see Acknowledgement). Criteria for inclusion were that the article or conference abstract evaluated birthweight as a possible risk factor for maternal urinary incontinence, with results presented in Norwegian, Danish, Swedish, or English The process for selecting studies is presented in Table 1. Information about origin, study design, response rate, number of participants, method of data collection, adjusted results, time of UI, mode of childbirth, BMI, weight of new-born, age, parity, and main findings for all included studies were extracted.

**Table 1:** Flowchart of included and excluded studies.

No reviews of this topic were identified. For obvious reasons, no randomized controlled studies (RCT) on birthweight and UI have been conducted. A considerable number of cohort studies and cross-sectional studies of high quality have been performed. Even though RCT’s provide the highest level of evidence, a summary of results from cohort studies and cross-sectional studies will be essential in order to evaluate a possible causal association between birthweight and UI.

### 2.1 Birth weight

Mean birth weight in Europe and USA [7] is approximately 3500 g. Weight cut-off at 3500 g and 4000 g gives information on the association between UI and birthweight beyond mean, as well as extreme birthweight, respectively. Weight cut-off on 3500 g and 4000 g were most common in identified studies, and were therefore chosen for this review.

Birthweight in one study was originally analysed according to the 50th and 90th percentile for birthweight (3,541 g and 4,180 g, respectively). These data have been re-categorized into 3500 g and 4000 g, and data has thereafter been reanalysed and stratified for mode of childbirth. Data were adjusted for BMI and weight loss after childbirth [8]. Birthweight in one study was categorized according to birthweight quartiles [9]; birthweight >3925 g from this study was included in analyses on the association between birthweight >4000 g and UI. Boyles et al used pounds [10]; birthweight of >8 lb. (3639 g) from this study was included in analyses on the association between birthweight >3500 g and UI.

### 2.2 Urinary incontinence

Information on UI after childbirth was categorized as questionnaires, objective testing, structured interviews, phone interviews, or reviews of existing medical records. No minimum cut-off for frequency, amount or severity of UI was set to be included in this article. Stress UI is more common after childbirth than urgency UI and mixed UI. The prevalence of pure stress UI is reported to be 2 – 8 times higher than the prevalence of pure urgency UI in pregnancy [1]. The stress/urgency ratio is reduced postpartum as prevalence of stress UI decline. Several studies focus solely on stress UI [[11], [12], [13], [14]], but few studies have data on urgency UI. Data on any UI was used in this meta-analysis. Separate analyses on available data on stress UI were also performed.

### 2.3 Assessment of quality and bias

Strengthening the Reporting of Observational Studies in Epidemiology (STROBE) Initiative has published recommendations on how to report data in cohort and cross-sectional studies [15]. STROBE was used to assess methodological quality. There was a high threshold to exclude studies. Studies with insufficient methodological information on study design, setting, statistical methods, and study participants were excluded from the systematic review and meta-analysis. Selection bias can affect the meta-analytic estimate. Selection bias was therefore considered in studies used in the meta-analyses, based on setting, study population and response rate. Risk of selection bias was considered as low, medium, high, and unclear. Information on studies included in meta-analyses regarding selection bias, adjustment of effect estimates, and reporting of all data (effect estimate, N in exposed and unexposed group, N with UI and continence) were collected. Funnel plot asymmetry based on standard error (log [OR]) was used to explore possible reporting biases.

### 2.4 Data synthesis

Birthweight was categorized as a dichotomous variable with two categories; <4000 g vs >4000 g, and <3500 g vs >3500 g, respectively. Available original data from included studies on birthweight (≥4000 g and ≥3500 g, respectively) and UI were extracted. Adjusted effect estimates were extracted when available. Unadjusted effect estimates were used when adjusted estimates were not presented; raw data and absolute numbers were then converted to unadjusted OR. Relative risk in one study was treated as OR [16]. To enable comparison across studies, Log [OR] with SE were calculated for each included study. Results were pooled and combined in meta-analyses. Estimates were inserted into Review Manager 5.3 for meta-analyses.

To reduce diversity in study characteristics, separate sub-group meta-analyses were performed according to type of UI, mode of delivery, primiparous women, and UI 3 – 18 months postpartum. Mode of delivery was categorized as any vaginal delivery or any CS. Main findings on the association between birthweight and UI are presented as odds ratio, and in Forest plot figures. Both adjusted data and unadjusted data are presented in Forest plot figures.

Heterogeneity among studies was assessed by I2. An I2 of 0% to 40% represents minimal heterogeneity, while 75% to 100% represents considerable heterogeneity. Adjusted data and unadjusted data had in general moderate to substantial heterogeneity in effect estimates. Random effect estimates were therefore used.

The review was registered in PROSPERO (73021); NHS’ International prospective register of systematic reviews. The review adheres to the PRISMA guidelines and MOOSE guidelines for meta-analyses and systematic reviews of observational studies.

## 3 Results

A total of 477 articles were identified. Fifteen external articles were added based on the authors’ knowledge of relevant literature, and after reading references. A total of 385 articles remained after removing duplicates (Table 1). Fifty-seven articles (N = 164,600) were included in this systematic review. Descriptive data are presented in Table 2. Twenty-two articles had data that could be included in the meta-analyses. Descriptive data are presented in Table 3.

**Table 2:** Descriptive data on studies included in the systematic review.

	Origin	Design	Asso- ciation	N	Data gathering	Respons-rate	Adjust.	Time point of UI	Birth-weight	Parity	Age	BMI	Delivery	Main finding
**Altaweel** [21] *	Saudi Arabia	Cross-sectional	++	2,180	Quest.: UDI-6, IIQ-7	30 %	Yes		> 4 kg	All	30 ± 10	All	SVD and CS	Birthweight of baby >4 kg OR 1.7 (1.4–2). Not data on type or severity by birthweight.
**Arya** [41]	USA	Cohort	-	315	Telephone interview and questionnaire. IIQ-7		Yes	2 weeks, 3 months, 1 year after delivery		Primiparous	21-23		SVD 48%	On univariate analysis, there was no association between the presences of urinary incontinence at any follow-up period after delivery infant birthweight. Hazard Ratio: 1,0 (95% CI 0,9-1,1)
													Forceps 29% Vacuum 24%	
**Baracho** [42]	Brazil	Cross-sectional	++	192	Delivery charts. Interview, ICIQ-SF, physical exam		Yes	5-7 mth pp	>2.988 kg	Primiparous	23,2	BMI>25: 39/192	SVD	Newborn weight (g), mean (SD) among women with UI: 3,206.4 (364.8). Among women without UI: 3,128.3 (372.8) 0.14*. Significant finding. Sign. association between SUI and weight >2.988 g
**Boyles** [10] *	USA	Cross- sectional	++	5,599	Quest.	39 %	Yes	3-6 mth pp	> 8 lb.	Primiparous, continent before pregn.	27	24	CS 27%. Forceps/vacuum 13%	Increasing UI with birthweight >8 lb., but only among women delivering by vaginal delivery: adjusted OR 1.22, (1.03–1.45). OR 0.84 (0.53–1.35) among women delivering by CS.
**Brown** [22] *	Australia	Cross- sectional	++	1,336	Statewide postal survey.	62 %	Yes	6-7 mth pp	>4 kg	All			SVD 69%. Forceps 11%. Emergency CS 9%. Elective CS 9%.	Infants weighing ≥ 4000 g associated with higher rates of urinary incontinence (37/196 [18.9%] *versus* 101/1097 [9.2%], OR 2.29 [95% CI 1.5-3.5]). Associations of assisted vaginal births controlling for duration of labor, birthweight of infant and perineal trauma. Infant birthweight: <3000 g, 3000-3999 g, >4000 g: Adjusted OR for UI: 1.90 [1.2-3.1]
**Burgio** [43]	USA	Cohort	-	523	Interview day 2. and 3, week 6 and 3, 6, and 12 mth pp.		Yes	6 weeks – 12 mth pp.		Mixed, parity 1,9	28,6			Heaviest previous birthweight OR 0.999, 95% CI 0.990- 1.008. p = 793
**Cardo** [44]	Spain	Cohort	-	272	Interviewed at term and 4 months pp. ICQ-SF and KHQ			4 mth pp		Mixed	31,8		SVD 62%. Forceps 4%. Vacuum 21%. CS 21%.	When only vaginal delivery is analyzed, no statistical association with newborn's weight was found.
**Caseym** [23] *	USA	Cohort	+	3,887	Interview	37 %	Yes	5-7 mth pp	> 4 kg	Primiparous	22,5	BMI 30		Univariate analyses: Birthweight >4000 g in 279 women (7%). Among these Urge UI 14 (9%, OR 1.3 (0.7- 2.3)). Stress UI 10 (7%, OR 1.0 (0.5- 1.9)). Adjusted analyses: association between stress UI and weight >4000 g: OR 1.2: 0.6 – 2.3.
**Castillo** [45]	Spain	Cohort	-	243	Quest; ICIQ-SF			6 mth pp.		Mixed	29,9	BMI 26,2	VD 66%.	No statistically significant differences were found between a worsening on quality of life and birthweight
**Chaliha** [46]	UK	Cohort	+	549	Interview, examination	100 %		3 mth pp	Mean 3.37 kg ± 0.49	Primiparous	29		SVD 53%. CS 24%	Fetal weight ass with urge UI: OR 11.3 95% CI 0.4- 352.8. Stress UI: OR 2.5 95% CI 1.1- 6.1
**Chou** [47]	Taiwan	Cross-sectional	-	378	Interview by telephone		Yes	1 year pp	Mean 3.116 kg	Primiparous	28,1	BMI 27,0	Vaginal 48%. CS 52%	Vaginal delivery: Birth body weight OR 0.999 (95% CI 0.997- 1.002, p = 0.543) for incident stress UI. When CS: Birth body weight OR 0.997 (95% CI 0.997- 1.002, p = 0.543) for incident stress UI (identical to vaginal delivery)
**Connolly** [48]	USA	Cross-sectional	-	3,205	Interview. Sandvik SI score >3.	36 %	Yes		> 4 kg	Mixed	49,2		Vaginal	There was an overall difference in the odds of moderate/ severe UI between the <4,000 g group, the ≥4,000 g group.
**Diez-Itza** [24] *	Spain	Cohort	-	376	Interview		Yes	6 weeks pp	> 4 kg	Mixed	32,4		Vaginal	Urgency only. Birthweight > 4 kg were not associated with **UUI** 6 weeks postpartum (OR 0.6, 95% CI 0.05 - 3.10)
**Diez-Itza** [17]	Spain	Cohort	-	272	Interview		Yes	2 years pp		Primiparous, continent before pregn.	31,2	BMI 23,4	Vaginal 86%. CS 14%.	Incident stress UI. No stress UI 2 years postpartum: Mean birthweight 3306. Stress UI 2 years postpartum: Mean birthweight 3281. P = 0.74.
**Dimpfl** [31] *	Germany	Cohort	-	350	Interview		No	6 and 12 weeks pp.	3.5 kg	Continent before pregn.			Vaginal 83%. CS 17%	Mothers, who gave birth to infants with a birthweight above 3500 g (permanent SUI: 4.7%) had no significantly higher incidence of pp UI than mothers with infants under 3500 g (7.0%). Chi’ = 0.22-n.s.
**Dolan** [32] *	UK	Cross- sectional	-/+	1,861	Sheffield Pelvic Floor Questionnaires.	62 %	Yes/No	20 years after delivery	Mean 3.285 kg	Parity 1,6	45,7	24,8	Vaginal 86%, CS 13,9	Adjusted OR for UI 12 years after delivery in primiparous < 3000 g OR 1.32 (0.73-2.39). 3000 – 3500 g Ref. > 3500 g OR 1.05 (0.59-1.86). Adjusted OR for UI 12 years after delivery in parous: < 3000 g OR 1.25 (0.96-1.62). 3000 – 3500 g Ref. > 3500 g OR 1.21 (0.97- 1.52).
**Eason** [25] *	Canada	Cohort	-	949	Quest. Info collected during a RCT of perineal massage during the 3. trimester.	79 %	Yes	3 mth pp	4 kg	Mixed	28,6		CS 18%	Baby's weight (g): <4000 N: 837 Risk: 31% OR 1.00. Baby's weight (g): ≥4000 N 112 Risk: 30% Crude OR 0.96 95% CI 0.73-1.48
**Eftekhar** [49]	Iran	Cohort	++ with frequen-cy	702	Quest. at prenatal visit week 28-29.	70 %		4 mth pp	3 kg	Primiparous continent before pregn.			Vaginal 51%. CS 49%	Stress UI. A birthweight greater than 3000 g appeared to be associated with increased frequency of SUI P = 0.000; x^2^ = 22.5.
**Emanuela** [50]	Italy	Cohort	-	93	Clinical examinations before delivery and at 3 and 6 months pp.	100 %		3 and 6 mth pp			32,6			Newborn weight did not show statistical differences in continent and incontinent patients
**Farrell** [37]	Canada	Cohort	-	484	Questionnaire and hospital charts.	83,50 %	No	6 weeks and 6 mth pp	Mean 3.489 kg	Primiparous	28		CS 25%. SVD 56%. Instrumental 19%.	Birthweight (kg) continent women: 3458 g, incontinent women: 3425 g, not significant difference.
**Frias** [51]	Spain	Cohort	+	89	Sandvik questionnaire, ICIQ-SF, PISQ-12			2 mth		53,7% primiparous	31,3	28,3	Eutocic 68%. Forceps 4% CS 28%.	More women with UI had babies >3000 g than women without…. 84% of women with UI had a newborn weight >3000 g compared with a rate of 60% of women without UI. No statistical differences.
**Fritel** [52]	France	Cohort	-	307	Questionnaire	46%	No	4 years	4 kg	Primiparous	29,3	21,3	CS 21% Forceps 36%	Univariate comparisons between women with current SUI and those with no SUI found no significant association between current birth weight
**Gartland** [26] *	Australia	Cohort	+	1,283	hospital records, quest and telephone interviews	28–31%	No	3, 6, 9, 12 and 18 mth pp	4 kg	Primiparous, continent before pregnancy	31		SVD 31%. CS 21%. Instr 32%.	Persistent UI 4 – 18 months postpartum. Birthweight were not significantly associated with persistent UI. Birthweight (g) <2500 OR 1.41Birthweight (g) 2500–3999 OR 1.0 (ref). Birthweight (g) ≥ 4000 OR 1.32.
**Glazener** [38]	Aberdeen; Scotland, Birmingham; England, Dunedin, NZ	Cross-sectional	++ for persistent UI starting in pregn.	3,405	Questionnaire survey in Maternity units and obstetric case note data	70-84%	Yes	3 mth pp	Mean 3.296 kg. Used quartiles.	Primiparous	26,7		SVD 58%, CS 17%, Instr 25%	Incontinence first occurring during pregnancy and still present at 3 months was associated with heavier babies (birthweight in top quartile, OR 1.56, 95% CI 1.12-2.19). Incident UI after delivery: < 3 kg Ref. 3.00–3.35 kg OR 1.26. 3.36–3.69 kg OR 1.42. ≥ 3.70 kg OR 1.33. Persistent UI starting during pregnancy: < 3 kg Ref. 3.00–3.35 kg OR 1.33. 3.36–3.69 kg OR 1.45. ≥ 3.70 OR 1.56.
**Grodstein** [53]	USA	Cohort(?)	-	83,168	Nurses’ health study		Yes	Late in life		All	60,4	20		For birthweight of the heaviest child, little association with UI. Somewhat lower risks with infant of >10.5 pounds at birth compared with <8.5 pounds. Risk for UI: <3.86 kg OR 1.00. 3.86-4.3 kg OR 1.03. 4.35-4.76 kg OR 1.05. > 4.76 kg OR 0.97.
**Groutz** [11] *	Israel	Cross-sectional	++	300	Interview	100%?		3 days p	3.5/4 kg	100 nulliparous. 100 primiparous. 100 ≥ para 5	20 - 43		VD only	No correlation between birthweight of the first newborn and prevalence of persistent, non-pregnancy-related stress urinary incontinence. Prevalence of persistent, stress UI among grand multiparous women delivering at least one baby >4000 g was 29.4%. Prevalence of persistent stress UI among grand multiparous whose newborns did not weigh more than 4,000 g was significantly lower (16.7%, P < 0.05).
**Groutz** [54]	Israel	Cross-sectional	-	363	Interview, hospital charts			1 year pp	mean	Primiparous continent before preg.	28-32	60-63 kg	SVD and CS	Birthweight among continent women: 3240 ± 408. Birthweight among incontinent women: 3330 ± 330. No significant difference.
**Gyhagen** [35]	Sweden	Cross-sectional	+	5,236	Questionnaire and birth registry	65 %	Yes	22 years pp	4.5 kg	Primiparous	50-53	26	VD 76% CS 24%.	Weight > 4 500 g compared to < 4 500 g among CS. OR 0.66 (95% CI 0.33–1.29). Weight > 4 500 g compared to < 4 500 g among VD 1.23 (95% CI 0.87–1.76). The risk of UI after VD vs CS increased with increasing birthweight.
**Hatem** [18] *	Canada	Cross-sectional	-	1,291	Questionnaire	52 %		6 months pp	4 kg	Primiparous	27,20	25,2	Mix	No association between birthweight > 4000 g and UI: OR 0.63 (0.30–1.31)
**Hvidman** [19] *	Denmark	Cross-sectional	-	376	Questionnaire	1 %	Yes	Few days pp and 3 mth pp		Mixed	29		CS 9%. Instr. 7%	Risk of UI first days PP and 6 mth pp OR 1.0 pr 500 g in adjusted analyses. Adjusted OR for UI first days PP 1.0. OR for UI > 4 weeks PP 1.2 (not sign). Adjusted OR for UI ≥ 12 weeks pp 1.1 (not sign).
**Iwanowicz** [12] *	Poland	Cross-sectional	++	313	Women treated for stress UI; medical history and urodynamic test.				4 kg	Mixed	50-53			The probability of the occurrence of SUI is statistically higher after vaginal delivery of a baby with birthweight of 4000 g or more. 45% of women with UI and 34% of women without UI had babies >4000 g (sign finding).
**Kashanian** [55]	Iran	Cohort	-	1,400	Questionnaire			1 year pp					VD 400. ECS 600. Acute CS 400	There was no significant difference between the women with SUI and without according to neonatal weight
**Koveleva** [9]	Russian Federation	Cohort	++ for mixed UI	518	Interview			4 mth pp	Mean	All	30,1		VD and CS	Mean weight of the newborn in group of patients with mixed UI was 3544 + 519 g, in the control group and 3173 + 740 g, (p < 0.01). A relative risk of occurrence mixed UI in group of women with weight of the newborn above 3544 + 519 g was higher (RR = 1,38; 95% CI - 1,02 to 1,85; p < 0,05).
**Krue** [13] *	Denmark	Cross-sectional	+	119	Questionnaire	89 %	No	6-30 mth pp	4 kg	Mix		>30	VD	Birthweight >4000 g vs <4000 g; stress UI 34% vs 31%, urge UI 6% vs 4%, mixed UI 15% vs 11%: p > 0.10. In the group whose infant birthweight was 4000 g or more the prevalence of stress incontinence 6–30 months postpartum was higher than in the <4000 g group (34.0% vs. 30.6%) (p > 0.10)
**Mallah** [20] *	Iran	Cohort	++	441	Examination, medical records		Yes	3 mth pp.	4 kg	Primiparous	28,1	31,5	Mix	The incidence of UI was higher in cases of vaginal delivery and birthweight greater than 4 kg. OR 4,8 (95% CI 3,0 - 7,7)
**Marsh** [56]	UK	Cross-sectional	-	324	Questionnaire			3 mth pp.	Mean 3.586 kg	82% primiparous			VD	Birthweight was not associated with increased risk of developing stress urinary incontinence
**McKinnie** [57]	USA	Cross-sectional	++	978	Questionnaire		Yes				42,7		VD	For each additional 16 ounces of infant weight delivered vaginally, the OR for UI increased by 1.13 (1.06–1.20).
**Obioah** [27] *	Nigeria	Cohort	++	230	Questionnaire interview		Yes	6 weeks, 3 mth pp		80% multipara, continent before pregn.	31,4		SVD 90%	Birthweight > 4 kg significantly associated with UI 3 months postpartum OR 5.60 (1.21–25.92)
**Rørtveit** [14] *	Norway	Cross-sectional	++	11,397	Questionnaire and birth registry	80 %	Yes		4 kg				VD	Significant associations between any UI and birthweight ≥ 4000 g (OR 1.1, 95% CI 1.0-1.2); moderate or severe incontinence OR 1.0 (0.9-1.2). ≥ 4000 g also associated with stress UI.
**Samuelsson** [28] *	Sweden	Cross-sectional	+	487	Questionnaire, gyno.examin.	76 %	Yes		Quar-tiles	Mixed	39	65 kg	Mixed	There were no significant correlations with birthweights >3925 g.
**Schytt** [16] *	Sweden	Cohort	++	2,390	Questionnaire Swedish Birth Register	53 %	Yes/no	1 year pp	3.5-4 kg	44% primiparous. Strati-fied on primiparous.	30,5		VD 79%. CS 13%. Instr 13%.	Birthweight >3500 g was associated with stress UI in multiparas (RR 1.4, CI 95% 1.1–1.7). Within the vaginal group: infant birthweight >3500 g (RR 1.3; CI 95% 1.1–1.6). There was no association in multivariate analyses. Some results are adjusted.
**Seshan** [58]	India	Cross-sectional	+	598	Questionnaire		Yes			Mixed	20-60			The weight of the largest baby delivered had the strongest impact on predicting UI symptom severity (UISS)
**Solans-Domenech** [29] *	Spain	Cohort	+	1,128	Questionnaire		No	7 weeks pp	> 4 kg	Continent, nulliparous women			CS 20%. VD 80%.	UI among 12/56 women with baby >4000 g, UI among 140/832 among women with baby <4000 g. Adjusted HR for incident UI postpartum among women with baby >4000 g: 2.8 (0.9–8.4)
**Thom** [30] *	USA	Retro-spective cohort	++	1,521	Questionnaire, interview, abstraction of labor and delivery records.		Yes		> 4 kg		56		VD	Weekly UI significantly associated with weighing 4,000 g or more (OR 1.47, 95% CI 1.16–1.86). When analyzed as a continuous variable, greatest birthweight showed evidence of a threshold effect with an increase in the risk of UI associated with increasing birthweight above about 3,200 g.
**Torkestani** [34]	Iran	Case-control	-	250	Questionnaire gyno.exam.		Yes			Mix	33-40		Mix	OR 0.928. 95% CI 0.43-2.00 for association with birthweight.
**Van Brummen** [59]	The Netherlands	Cohort	- -	344	Questionnaire	723 %	Yes	3 and 12 mth pp	3,418 vs 3,549	Nulliparous	30-31	21-26	VD 83%. CS 17%.	Infant birthweight 3,418 vs 3,549 as risk factor for urgency 1 year pp among women delivering by VD: adjusted OR 0.9 (0.98–0.99). No association was found for stress UI or urge UI.
**Viktrup** [60]	Denmark	Cohort	+	305	Questionnaire			12 mth pp		Mix			VD 82%. CS 18%.	Birthweight was increased in infants of mothers who developed stress UI after deliver, but not significantly: p = 0,07
**Volloyhaug** [61]	Norway	Cross-sectional	+	1,641	Questionnaire		Yes	Mean 20 years		Parous, mean 2,3	47	25,8	VD 42% OD 42% CD 14%	Parity and the largest infant’s birthweight were additional independent risk factors for UI but did not remain signiﬁcant in a multivariable logistic regression analyzes.
**Wesnes** [62] *	Norway	Cohort	++	5,219	Questionnaire, birth registry	45 %	Yes	6 mth pp	50/ 90 percentile. Re-analyzed on 3,5/4 kg	Primiparous continent before- and during pregn.	27	23,6	SVD only	Baby's birthweight between the 50th - 90th percentile (3541 - 4180 g) and > 90th percentile (> 4,180 g) were statistically significant risk factors for incident UI 6 months postpartum (OR 1.4; 95% CI 1.2 - 1.6 and OR 1.6; 95% CI 1.2 - 2.0, respectively) as compared to birthweight below the 50th percentile. Data reanalyzed for 3500 g and 4000 g.
**Williams** [63]	UK	Retro-spective, cross-sectional	++(stress) -- (urge)	482	Questionnaire	23 %		12 mth pp.						Birthweight was associated with incident stress UI (spearman r coefficient r = 0,04) and protective on incident urge UI (r coefficient r= - 0,04)
**Wu** [64]	China	Cross-sectional	+ +	2,500	Interview						43,5		Mix	Fetal weight was associated with stress UI only OR 1,64 (95% CI 1.27–2.13), p < 0,001 for macrocosmic infant compared with normal birthweight
**Yang** [65]	China	Cohort	-	1,889	Telephone interview		Yes	6 mth pp		Primiparous	30,6	72,9 kg	VD 45%. CS 55%.	No association between neonate birthweight and SUI, UUI or MUI.
**Yohay** [66]	Israel	Cohort	-	37	Questionnaire medical records, telephone interview	32 %		3 mth pp	3.344 kg	Multiparous mean 2,7	30,8		SVD 73% CS 23% OD 4%	Other obstetrical parameters including episiotomy and birthweight were not found to be significantly associated with any of the PFD items.
**Yip** [67]	Hong Kong	Cohort	-	148	Telephone interview			4 years pp.	3.2 kg	Nulliparous, continent before pregn.	27-28		VD 100%	The logistic regression analysis showed that birthweight was not significantly associated SUI 4 years after the index pregnancy-
**Zanelli** [68]	Italy	Cohort	++	452	Questionnaire			3 and 12 mth pp						Statistical correlation with incontinence 3 months postpartum was found for high fetal weight
**Zhang** [69]	China	Cross-sectional	++	4,684	Questionnaire	72 %	Yes			1,1	40	21,9	VD 80% CS 20%.	A multiple logistic regression analysis showed fetal birthweight was common potential risk factors for LUTS (OR 1.40, 1.07–1.85), voiding (OR 1.42, 1.08–1.87) and storage symptoms (OR 1.63, 1.16–2.28).
**Zhu** [70]	China	Cross-sectional	-	5,221	Interview	?	Yes							Birthweight was not identified as potential risk factors of female SUI.

Preg = pregnancy. PP = postpartum. Quest = questionnaire. SVD = spontaneous vaginal delivery. CS = cesarean section. VD = vaginal delivery. Instr = instrumental delivery. OD = operative delivery. Adj. = adjusted analyses. OR = odds ratio. RR = relative risk. Mth = months. UI = urinary incontinence. SUI = stress urinary incontinence. UUI = urgency urinary incontinence. MUI = mixed urinary incontinence. OR = odds ratio. ++ = significant positive association between birthweight and UI. + = non-significant positive association between birthweight and UI. - = non-significant negative association between birthweight and UI. -- = significant negative association between birthweight and UI. * = studies used in meta-analysis.

**Table 3:** Descriptive data on studies included in meta-analyses.

Study	Data on 4000 g	Data on 3500 g	Data on stress UI	Data 3-18 months postpartum	Data on vaginal delivery	Data on primi-parous	Risk of selection bias	Adjusted effect estimates
Altaweel [21]	X						Low	X
Boyles [10]		X (8 lb)		X	X	X	Low	X
Brown [22]	X			X			Low	X
Casey [23]	X		X	X		X	Low	For stress UI
Diez-Itza [24]	X				X		High	X
Dimpfl [31]		X	X	X			Low	
Dolan [32]		X				X	Low	Partly
Eason [25]	X			X			Low	
Gartland [26]	X			X		X	Low	
Groutz [11]	X		X		X		Low	
Hatem [18]	X			X		X	Moderate	
Hvidman [19]	X			X			Moderate	X
Iwanowicz [12]	X		X		X		Unclear	
Krue [13]	X		X		X		High	
Mallah [20]	X			X		X	Unclear	X
Obioah [27]	X			X			Low	X
Rørtveit [14]	X		X		X		Low	X
Samuelsson [28]	3925						Low	
Schytt [16]		X	X	X	X	X	Low	
Solans-Domenech [29]	X					X	Low	
Thom [30]	X				X		Low	X
Wesnes [62]	X	X		X	X	X	Low	X

UI = urinary incontinence.

Selection bias was considered in studies used in the meta-analyses. Risk of selection bias was considered as high in 2/22 studies [13,17], moderate in 2/22 studies [18,19], unclear in 2/22 studies [12,20], and low in 16/22 studies (Table 3). Unadjusted association between birthweight and UI was reported in 9/22 studies. Funnel plot did not reveal publication bias, as it spread evenly on both sides of the average, creating a roughly funnel-shaped distribution.

There was a significant positive association between high birthweight and UI after childbirth in 35% (20/57) of the studies. There was a non-significant positive association in 19% (11/57) of the studies. There was no association in 46% (26/57) of the studies. A significant protective association between high birthweight and urgency UI was also found in one of the above studies.

### 3.1 Birthweight >4000g

**Fig. 1:**
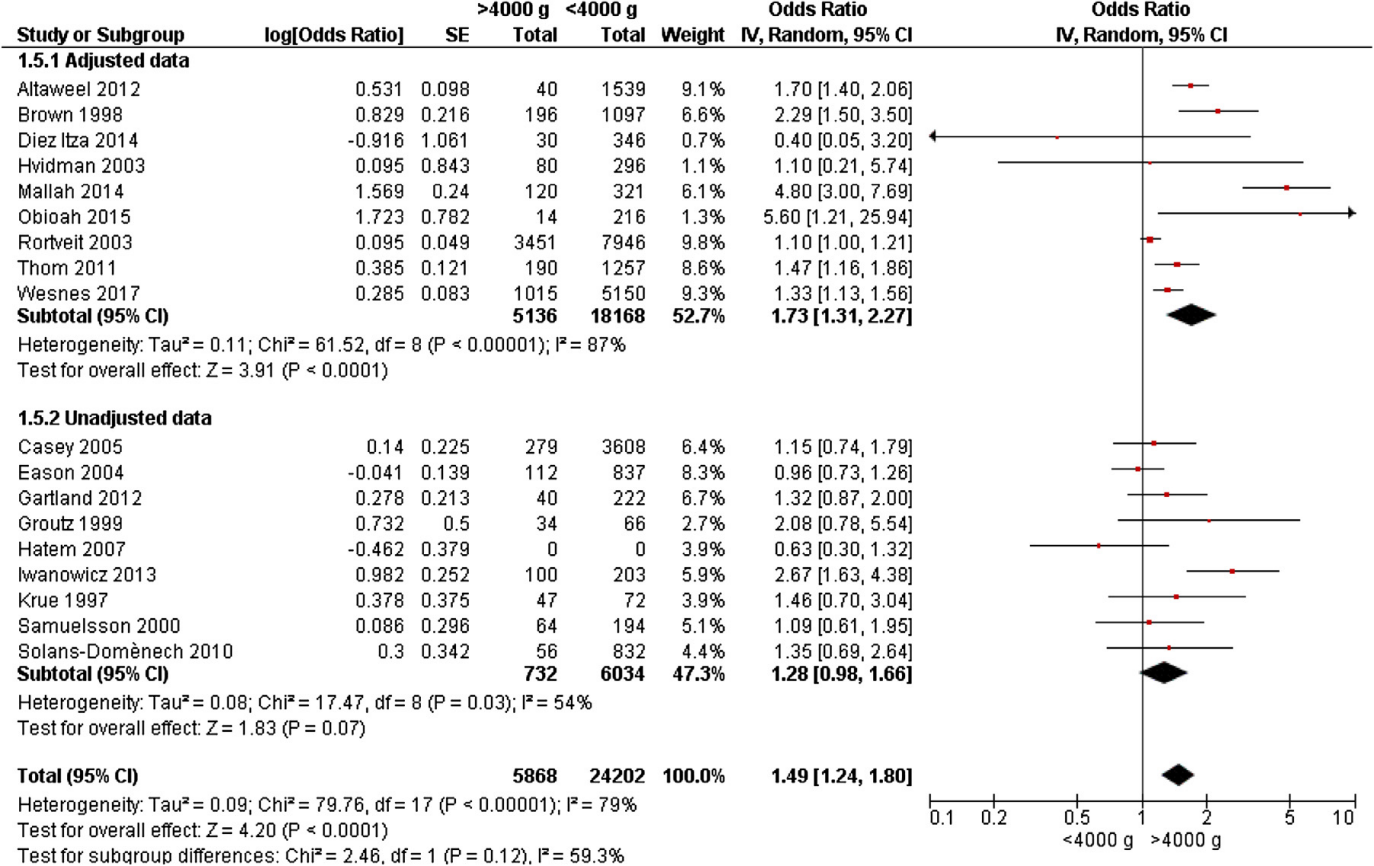
Forest plot of the association between urinary incontinence and birthweight >4000 g vs <4000 g, stratified for adjusted and unadjusted data.

Eighteen studies [8,[11], [12], [13], [14]] [[18], [19], [20], [21], [22], [23], [24], [25], [26], [27], [28], [29], [30]], reported data on 30,070 women for review on birthweight >4000 g and UI. Birthweight >4000 g compared to weight <4000 g was associated with a significantly increased risk of any UI in meta-analyses (OR 1.49, 95% CI 1.24 – 1.80) (Fig. 1). Meta-analyses revealed higher OR of UI in adjusted data than unadjusted data (OR 1.73 and OR 1.28, respectively). Funnel plot did not reveal publication bias, as it spread evenly on both sides of the average, creating a roughly funnel-shaped distribution.

#### 3.1.1 Birthweight >4000 g and stress UI

Data from four European [[11], [12], [13], [14]] and one American [23] study were available for meta-analyses. Time of recording UI varied from 3 days postpartum [11] to several decades after childbirth 14] Weight >4000 g was associated with a significant increased risk of stress UI (OR 1.52, 95% CI 1.03 – 2.25) when analysing available data from these five studies with a total of 15,806 women.

#### 3.1.2 Birthweight >4000 g and UI 3 – 18 months postpartum

**Fig. 2:**
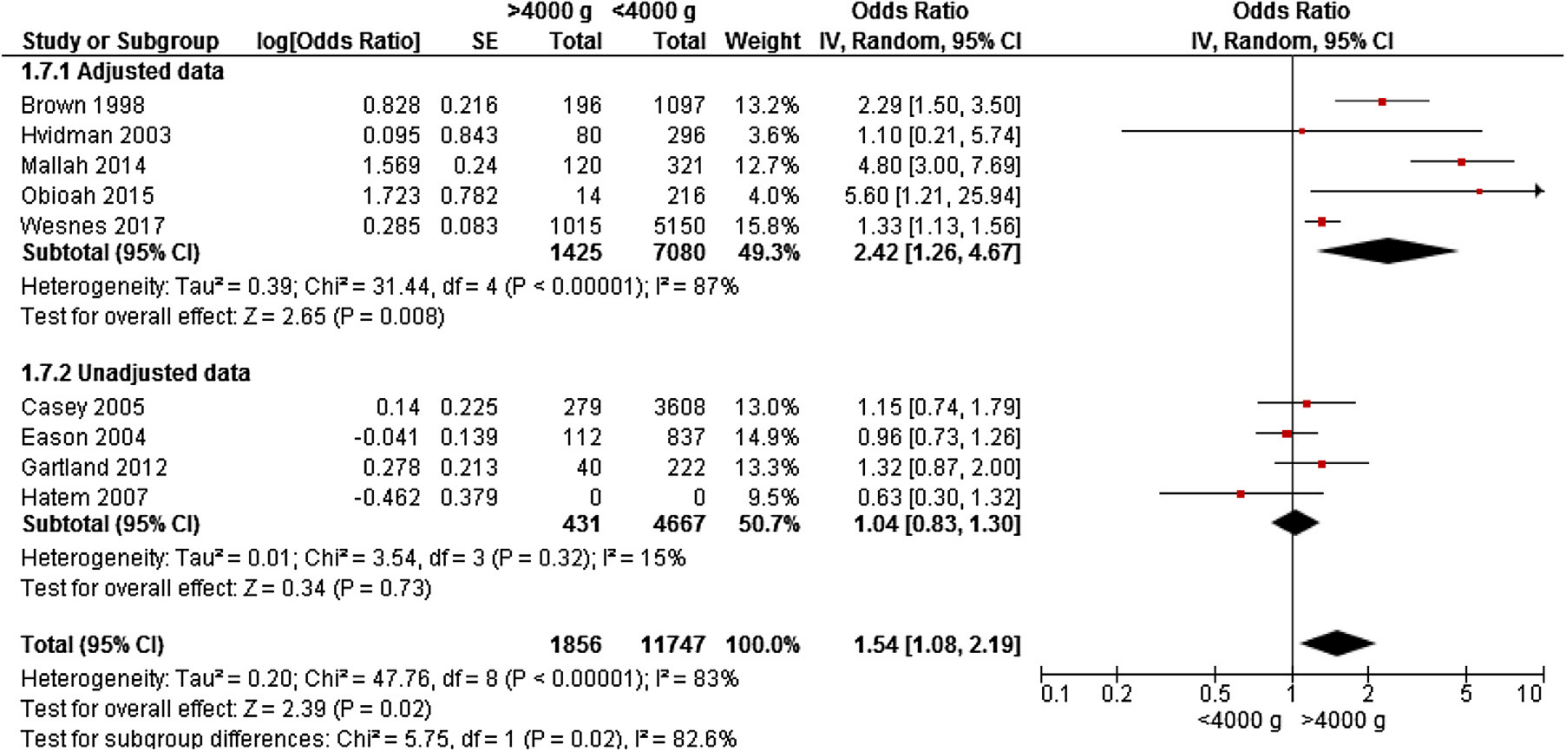
Forest plot of the association between urinary incontinence 3 – 18 months postpartum, and birthweight >4000 g vs <4000 g, stratified for adjusted and unadjusted data.

Nine studies; six cohort studies [8,20,23,[25], [26], [27]] and three cross-sectional studies [18,19,22] gave data on 13,603 women for meta-analyses. The studies were conducted in Europe [8,19], Africa 27], Asia^20^], Australia^22^,^26^], and America[18,23,25]. Age was 22 – 31 years in the cohorts; two cross-sectional studies reported age 27 – 29 years. Five studies included primiparous only [8,[18,20,23,26]. Birthweight >4000 g lead to a significantly increased OR 1.54 (95% CI 1.08 – 2.19) for UI 3 – 18 months postpartum compared to women delivering infants with birthweight <4000 g (Fig. 2).

#### 3.1.3 Birthweight >4000 g and UI after vaginal birth

**Fig. 3:**
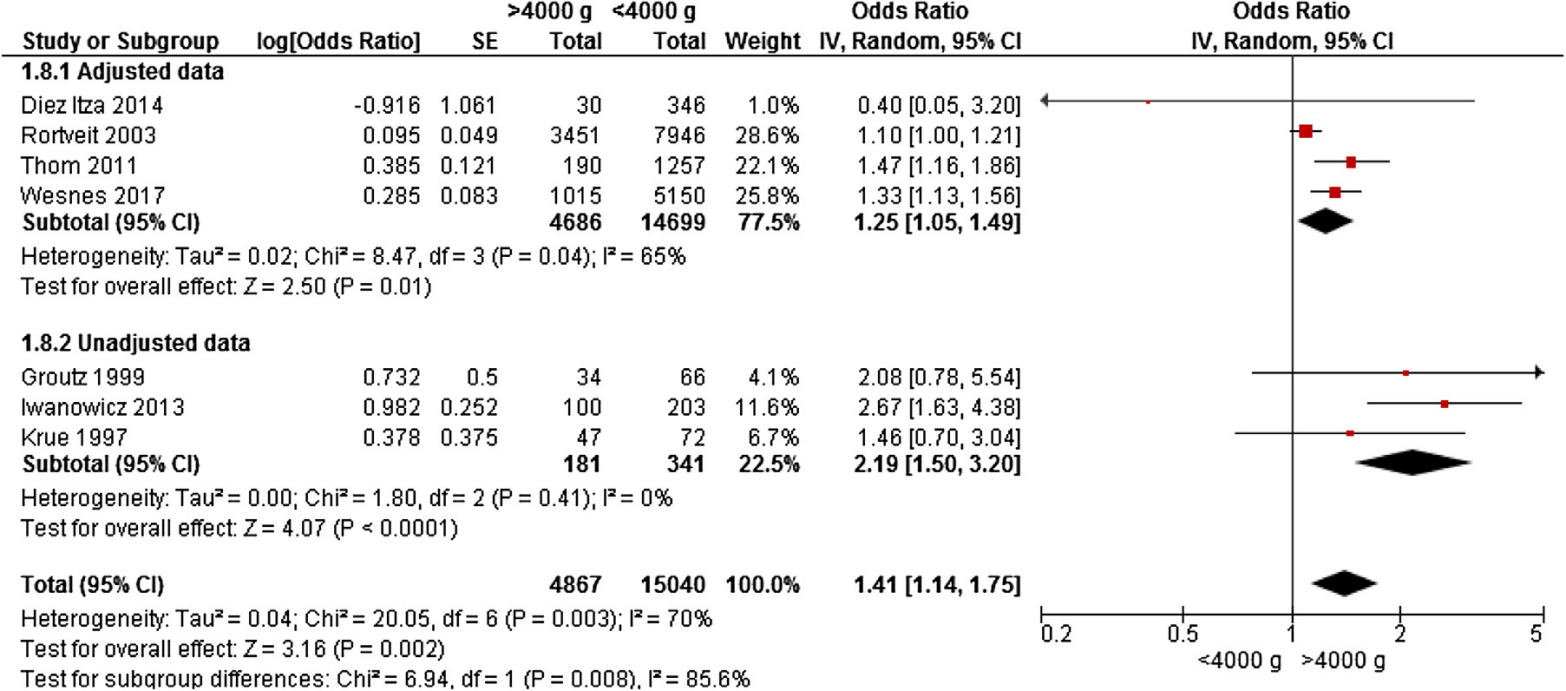
Forest plot of the association between urinary incontinence after any vaginal delivery, and birthweight >4000 g vs <4000 g, stratified for adjusted and unadjusted data.

Seven studies gave data for meta-analyses on 19,907 women on the association between UI and birth weight >4000 g among women delivering by any vaginal birth; three cohort studies [8,24,30] and four cross-sectional studies [[11], [12], [13], [14]] were identified. Three large studies with adjusted data were included; Rortveit et al.^14^] enrolled 11,397 women; Wesnes et al. [8] enrolled 5,219 women, and Thom et al. enrolled 1,521 women. However, mean age, parity and time of UI varied in these studies. Weight >4000 g was associated with a significantly increased risk of UI after vaginal birth (OR 1.41, 95% CI 1.14 – 1.75) (Fig. 3).

Only one study had additional data on birthweight >4000 g and birth by CS [8]. OR for UI after birth by any CS of child >4000 g compared to <4000 g was 1.38 (95% CI 0.84 – 2.28).

#### 3.1.4 Birthweight >4000 g and UI among primiparous women

Five cohort studies [8,20,23,26,29] and one cross-sectional study [18] had data on 11,643 women for meta-analyses on birthweight >4000 g among primiparous women. All studies had data on UI 2 – 18 months postpartum. Three studies included only women who were continent before pregnancy [8,26,29]. Weight >4000 g was associated with a non-significantly increased risk of UI among primiparous women (OR 1.46, 95% CI 0.95 – 2.26). Only two studies gave adjusted effect estimates [8,20], leading to an OR of 2.48 (95% CI 0.70 – 8.71) for UI among primiparous women delivering babies >4000 g compared to <4000 g. However, due to heterogeneity in effect estimates, I2 was 96%. Unadjusted analyses gave OR of 1.15 (95% CI 0.88 – 1.50).

### 3.2 Birthweight >3500 g

**Fig. 4:**
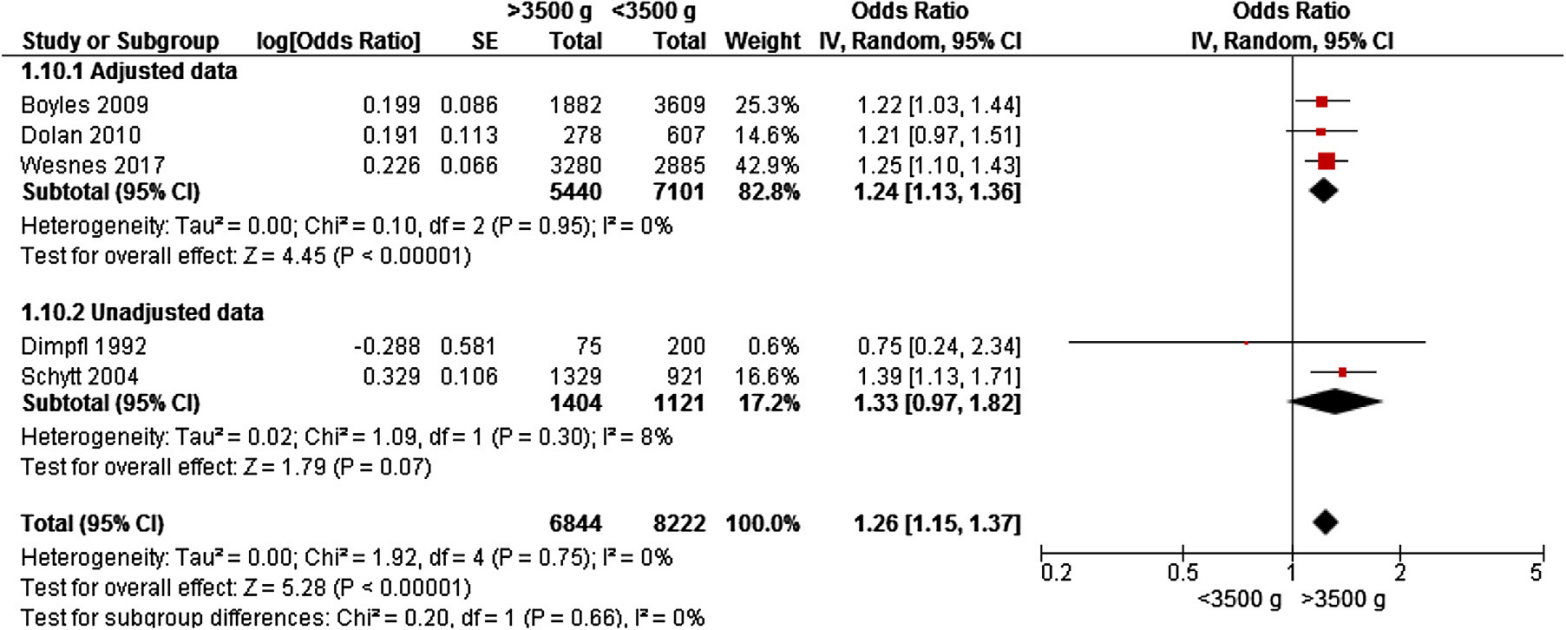
Forest plot of the association between urinary incontinence and birthweight >3500 g vs <3500 g, stratified for adjusted and unadjusted data.

Only four studies from Europe [8,16,31,32] and one from America [10] gave data on birthweight >3500 g and risk of any UI, including 15,066 women for meta-analyses. Two studies reported data on primiparous women[8, [10]; three studies included women who were continent before pregnancy [8,[10,31]. Weight >3500 g was associated with a significantly increased risk of UI (OR 1.26, 95% CI 1.15 – 1.37) (Fig. 4).

#### 3.2.1 Birthweight >3500 g stress UI

Two studies had unadjusted data on the association between birthweight >3500 g and stress UI [16,31]. Data was collected 6 weeks – 1 year after childbirth. None of these studies reached statistical significance on the association between birthweight >3500 g and stress UI. Birthweight >3500 g was associated with a non-significantly increased risk of stress UI in meta-analyses of 2525 women (OR 1.33, 95% CI 0.97 – 1.82).

#### 3.2.2 Birthweight >3500 g and UI 3 – 12 months postpartum

**Fig. 5:**
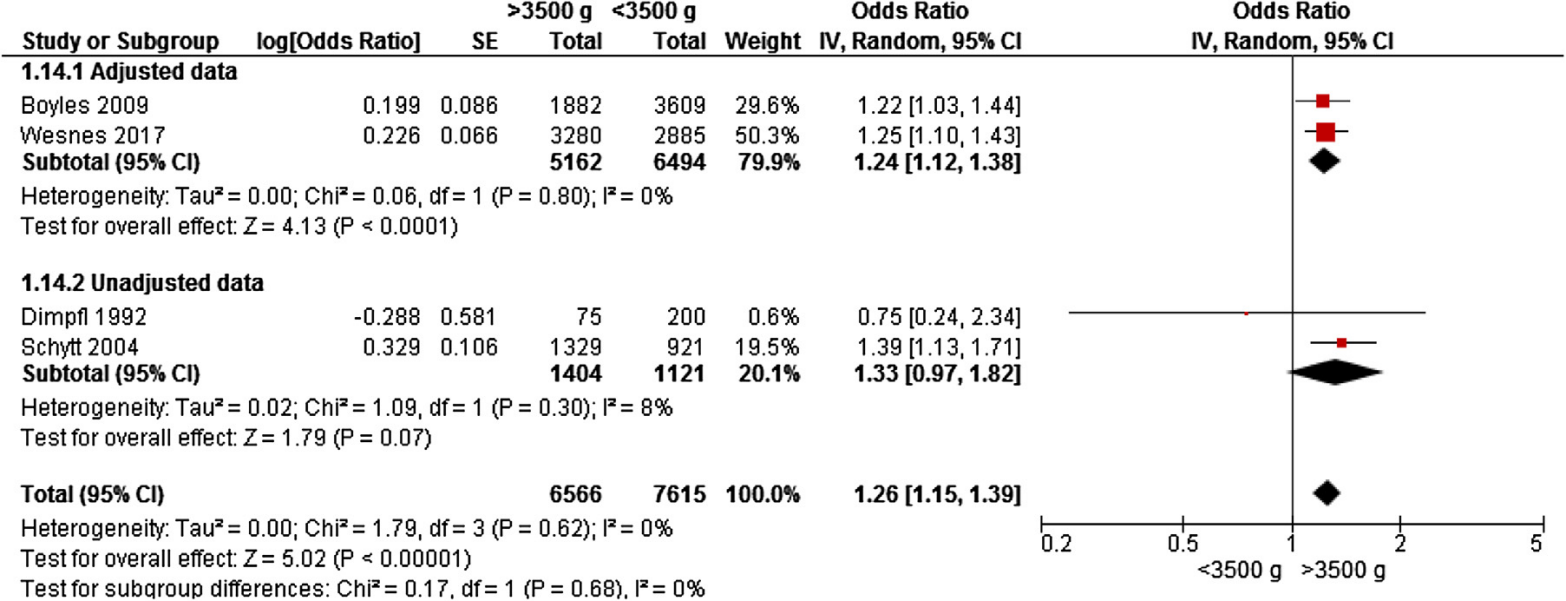
Forest plot of the association between urinary incontinence 3 – 12 months postpartum and birthweight >3500 g vs <3500 g, stratified for adjusted and unadjusted data.

Four studies reported data on birth weight >3500 g and UI 3 – 12 months after childbirth [8,10,16,31]. Included studies were rather similar regarding study population; three studies reported data on primiparous [8,10,16], and three studies reported data on women who were continent before pregnancy [8,10,31]. Meta-analyses on 14,181 women found a significantly increased risk of UI 3 – 12 months postpartum (OR 1.26, 95% CI 1.15 – 1.39) (Fig. 5).

#### 3.2.3 Birthweight >3500 g and UI after vaginal childbirth

Three large studies with 5599 [10], 2390 [16], and 5219 [8] participants had data on the association between birthweight >3500 g and UI after vaginal childbirth. All studies presented adjusted data on UI 3 – 12 months postpartum with OR 1.22, 1.30 and 1.25, respectively. In meta-analyses, weight >3500 g was associated with a significantly increased risk of UI after vaginal childbirth (OR 1.26, 95% CI 1.15 – 1.37). I2 was 0%.

Boyles [10] and Wesnes [8] reported stratified data for CS: there was no association between birthweight >3500 g and UI after CS (OR 1.04, 95% CI 0.67 – 1.63).

#### 3.2.4 Birthweight >3500 g and UI among primiparous women

Four large questionnaire-based studies investigated the association of birthweight >3,500 g on UI [8,10,16,32] in women 3 – 12 months after childbirth. Three studies reported adjusted results. Two studies found a significant positive association [8,10] between birthweight >3,500 g and UI among primiparous women, two studies found a non-significant positive association 16,32]. Meta-analyses from these studies gave an OR of 1.23 (95% CI 1.11 – 1.35) for UI among primiparous women delivering infants with birthweight >3500 g compared to <3500 g.

Schytt et al [16] reported stratified data on primiparous and multiparous women. Results indicated that birthweight >3500 g lead to higher RR for UI postpartum among multiparous compared to primiparous women (RR 1.5 (95% CI 1.2–1.9) and RR 1.1 (95% CI 0.8–1.4), respectively).

## 4 Discussion

This is the first systematic review on the association between birthweight and UI after childbirth. UI postpartum is a common condition, affecting 32 – 36% of women [2]. Many risk factors have been brought forth in studies, most are not well documented. Reviews on epidural [3], episiotomy [4], cesarean section [2] and instrumental childbirth [5] have made their association with UI postpartum clear. Some birth variables are more commonly extracted from birth registries (birthweight, head circumference, rupture) and often included in analyses. There has been need to summarize knowledge on these potential risk factors.

### 4.1 Main findings

Birthweight >4000 g and >3500 g were associated with significantly increased risk of UI (OR 1.49 and 1.26, respectively). Separate analyses on stress UI, UI 3 – 18 months postpartum, UI after vaginal birth and UI among primiparous women also revealed significantly increased risk of UI.

### 4.2 Strengths and limitations

Prevalence of UI after childbirth varies with time of information gathering, type of UI, mode of childbirth, and characteristics of the study population [1]. Diversity in studies included in meta-analyses needs to be addressed. To control for some of these parameters, separate meta-analyses were performed for time of UI, stress UI, vaginal birth, and parity. Subgroup meta-analyses on the reported variables showed significantly increased OR in the range 1.41 – 1.54 for UI and birth weight >4000 g. Corresponding analyses on UI and birth weight >3500 g gave significantly increased OR in the range 1.23 – 1.33. This indicates that the overall risk estimates are robust. Studies have shown that selection bias affects prevalence estimates, but data are still valid for risk estimates [33]. High birthweight is also associated with high BMI in the mother, prolonged birth, CS, rupture, episiotomy, birth by forceps and vacuum. We cannot rule out confounding.

A total of 33/57 studies report on the association between birthweight and UI as secondary finding, without authors reporting values, percentages or risks. Information from these articles was applicable for this review, but not for the meta-analysis.

The literature search also needs to be addressed. Birth weight and UI was the main objective in few articles. Literature search retrieved information from headings and abstracts, therefore it did not retrieve articles where relevant information was solely in the main text. 476 articles were identified by literature search. Author’s knowledges of literature, and full reference reading identified 15 external articles (Table 1). A total of 27 articles were not evaluated for inclusion due to foreign language or lack of access. We must therefore accept that the sensitivity of the search was high, but not complete. Publication bias may be a problem, as significant data are more likely to be published, and presented in abstracts.

Categorizing of weight groups and reference groups can affect the results. Birthweight >3500 g and > 4000 g led to OR 1.26 and 1.49, respectively. The reference groups were birthweight <3500 g and < 4000 g, respectively. One study with 5,219 women used lower reference value [8]. OR of UI was 1.6 (95% CI 1.2 – 2.0) after birthweight >4,180 g (90 percentile) compared to birthweight 3,540 g (50 percentile) [8]. Another study compared birthweight >3,925 to <3,199, finding OR of 1.4 [28]. Risk estimates in studies might have been higher if reference groups consisted of lower birthweights.

Few studies use weight groups >4500 g [16,34,35]. Few study participants in the exposure group makes it difficult to do meta-analyses. One study reported adjusted OR 1.23 (95% CI 0.87 – 1.76) for UI among women delivering babies with birthweight >4,500 g compared to < 4,500 g 35].

Vaginal birth is associated with higher risk of UI than CS in most studies [2]. Weight >4000 g was associated with UI after vaginal birth and CS (OR 1.41 95% CI 1.14 – 1.75 and OR 1.38, 95% CI 0.84 – 2.28, respectively). Weight >3500 g was also associated with UI after vaginal birth and CS (OR 1.26, 95% CI 1.15 – 1.37, and OR 1.04 95% CI 0.67 – 1.63, respectively). These meta-analyses on birthweight and UI are in line with what is previous shown regarding birth mode and UI [2]; lower risk of UI after CS, than after vaginal birth. Studies including women delivering by vaginal birth and CS indicate that birthweight is a significant risk factor for UI only in association with vaginal birth [8,35]. Mode of birth is a confounder that is likely to affect the association between birthweight and UI in the remaining sub-analysis. There were, however, no data available for further stratified sub-analysis on CS and vaginal birth.

It is unclear how high birthweight lead to UI. Trauma to the pelvic floor when delivering large babies is a plausible contributing factor. Heavier babies have been associated with EMG evidence of pudendal nerve damage in the pelvic floor after vaginal birth [6,36].

The majority of studies report grams, others report pounds [10]. Several studies analysed on mean birthweight [37], some use percentiles [8] or quartiles [38]. Birthweight was often categorized arbitrary without authors reporting reason for cut-off, most studies uses 500-gram groups (3500 g, 4000 g, and 4500 g). Varying reporting and different weight categorizing makes it difficult to summarize published results. Weight cut-off on 3500 g and 4000 g were most common, and were therefore chosen for this review. As 3500 g generally represents mean birth weight, these weight cut-offs gives important information on the association between UI and birthweight beyond mean, as well as extreme birthweight >4000 g.

### 4.3 Interpretation

Birthweight appears to be a risk factor for UI after childbirth. Data in this meta-analysis are gathered from epidemiological cohort and cross-sectional studies. When planning future epidemiological studies, data on birthweight ought to be gathered when possible. Birthweight is likely to effect risk estimates on UI after deliver, and adjustment should be considered in future research.

The Hill Criteria are useful when considering a possible causal relationship between exposure and outcome. Birthweight satisfies several of the Hill Criteria for causation; it is biologically plausible, exposure precedes outcome, and there is consistency in the majority of studies. There is a dose-response association, as birthweight >4000 g gave higher OR for UI than >3500 g.

To predict future risk of UI after childbirth, the UR-CHOICE risk calculator intend to include eight variables, of which birthweight is the only fetal factor [39]. Birthweight is included in the risk calculator due to the high likelihood of causation.

Even though results appear biological plausible, results in the meta-analysis are based on epidemiological research and can thereby not automatically be applied into a clinical setting. As high birthweight is associated with UI postpartum, clinical preventive strategies ought to be identified. Strategies might be targeted on identifying mothers at risk of having babies with high birthweight (for instance by identifying high maternal BMI, identifying previous deliveries of babies with high birthweight), avoiding high birthweight (for instance by avoiding high maternal weight gain during pregnancy, detecting gestational diabetes), detecting high birthweight (for instance by growth charts, ultrasound, symphysis-fundus height measurements), reducing risk of incident UI postpartum (for instance by aiming at normal weight before pregnancy, and at regaining pre-pregnancy weight postpartum, considering CS, performing PFMT), or by treating UI postpartum, for instance by PFMT. Future research will need to find ways to identify which women are likely to give birth to babies >3500 g, and look into the best preventive strategies.

Pelvic floor muscle training is generally recommended in pregnancy and postpartum. It has been unclear which women benefit the most from this training. Women delivering babies with birthweight > 3500 g are women at risk of developing UI. No preventive strategy is validated in this study, but pelvic floor muscle training has documented effect on preventing UI among women delivering heavy babies [40].

## 5 Conclusion

We conclude that birthweight appears to be a risk factor for UI after childbirth. A causal relationship between birthweight and UI is biologically plausible. Strategies towards preventing UI postpartum should be targeted towards women at higher risk, like women giving birth to babies with high birthweight.

## Author contribution to the manuscript

Wesnes: Project development, data collection, manuscript writing, statistical analysis.

Seim: Data collection, manuscript writing.

## Funding, financial disclaimers

None.

## Declaration of Competing Interest

The authors report no declarations of interest.
